# Supplementary material for: Content-rich biological network constructed by mining PubMed abstracts
Source: BMC Bioinformatics. 2004 Oct 8;5:147. doi: 10.1186/1471-2105-5-147 (PMC528731; doi:10.1186/1471-2105-5-147)
Supplement: Additional File 2 — The original results of the above study (non-essential files are deleted to keep the file size under the limit set by BMC bioinformatics). [file 1471-2105-5-147-S2.bz2 › chilibotAdditionalFile2/dip05/218ID9694899E726/html/IL6R_IL6.html]

 


 **IL6R** and **IL6** 
  
Found 377 abstracts in PubMed, retrieved 05.  
 

 What does Google say? 
 PDF only 
| .edu only 

---

**Interactive relationship** (e.g. stimulation, inhibition, etc)

**Non-interactive relationship** (e.g. studied together, co-existance, homology, etc.)

- Aim To measure serum concentrations of the soluble interleukin 2 receptor sIL2R, soluble interleukin 4 receptor sIL4R, soluble interleukin 6  [ **IL6** ]  receptor  [ **IL6R** ]  sIL6R, and soluble tumor necrosis factor alpha receptor I and soluble tumor necrosis factor alpha receptor II, during the perinatal and early neonatal period, in order to evaluate their role in activation of immune response in labor and the first days postpartum.  Ref: 12857603 Mediators Inflamm, 2003
- Cellular cholesterol depletion triggers shedding of the human interleukin 6  [ **IL6** ]  receptor  [ **IL6R** ]  by ADAM10 and ADAM17 TACE.  Ref: 12832423 J Biol Chem< MedlineTA>J Biol Chem, 2003
- Increased soluble interleukin 6  [ **IL6** ]  receptor  [ **IL6R** ]  in vitreous fluid of proliferative vitreoretinopathy.  Ref: 12789531 Curr Eye Res, 2003
- We have determined the percentage of alphabeta and gammadelta T cells by flow cytometry as well as serum interleukin 6  [ **IL6** ]  IL 6  [ **IL6** ]  and soluble interleukin 6  [ **IL6** ]  receptor  [ **IL6R** ]  sIL 6R levels by enzyme linked immunosorbent assay in kidney allograft recipients with acute, chronic or stable graft evolution.  Ref: 12828564 Scand J Immunol< MedlineTA>Scand J Immunol, 2003
